# Supplementary material for: Imaging spatiotemporal evolution of molecules and active sites in zeolite catalyst during methanol-to-olefins reaction
Source: Nat Commun. 2020 Jul 20;11:3641. doi: 10.1038/s41467-020-17355-6 (PMC7371645; doi:10.1038/s41467-020-17355-6)
Supplement: Supplementary file 1 — Supplementary Information [file 41467_2020_17355_MOESM1_ESM.pdf]

Supplementary Information

Imaging spatiotemporal evolution of molecules and active sites in zeolite catalyst during methanol-to-olefins reaction

Gao *et al.*

Table of Contents

Supplementary Note 1: Characterization of SAPO-34 zeolites

Supplementary Note 2: Experiments and reaction-diffusion simulations of MTO reaction

Supplementary Note 3: Characterization of acidity and retained carbonaceous species

Supplementary Note 4: Time-dependent density functional theory

Supplementary Note 5: Super resolution structured illumination microscopy

Supplementary Note 6: Molecular dynamics simulations

Supplementary Note 7: Detailed results of reaction-diffusion simulations during MTO reaction

Supplementary Note 1: Characterization of SAPO-34 zeolites

All SAPO-34 zeolite samples were calcined at 873 K for 10 h by air to remove the organic template. The powder X-ray diffractions (XRD) signals were recorded for all samples using a PANalytical X'Pert PRO X-ray diffractometer with Cu Kα radiation (λ = 1.5418 Å) operating at 40 kV and 40 mA. As shown in Supplementary Figure 1, the XRD diffraction signals coincide with that for **CHA** topology, and all samples are free from impurity phases.

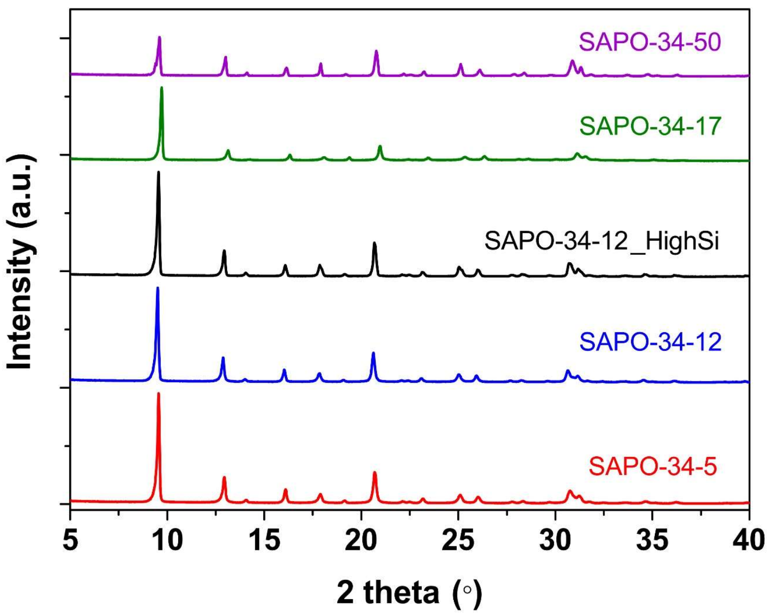

Supplementary Figure 1: XRD signals for calcined SAPO-34-5, SAPO-34-12, SAPO-34-12_HighSi, SAPO-34-17 and SAPO-34-50 zeolite samples.

We controlled the crystal size of SAPO-34 zeolite samples by regulating synthetic formulation before experiments[1]. Supplementary Figure 2 shows the field emission scanning electron microcopy (FE-SEM) images of SAPO-34 zeolites, and, as can be seen, all samples exhibit similar cubic morphology. The crystal size distributions of SAPO-34 zeolite crystals were monitored by FE-SEM, as shown in Supplementary

Figure 2. The number of crystals used for statistics of crystal size distribution is about 80 ~ 120 for each measurement. We found that the crystal size is 4.82 ± 0.36 μm for SAPO-34-5, 11.17 ± 1.80 μm for SAPO-34-12, 11.68 ± 1.22 μm for SAPO-34-12_HighSi, 16.92 ± 1.66 μm for SAPO-34-17 and 47.08 ± 3.50 μm for SAPO-34-50. The maximum deviations of crystal size for all SAPO-34 zeolite samples were controlled within 15%. The size distributions of all SAPO-34 zeolite samples are relatively uniform. The upper and lower limit of crystal size of SAPO-34 zeolite samples were then separately used as input parameters of the model to examine the effect of size distribution on simulated results.

Energy dispersive X-Ray spectroscopy (EDX) was analyzed by a FE-SEM Hitachi SU8020 equipped with a Horiba X-max silicon drift X-ray detector. X-rays fluorescence (XRF) was measured by a Philips Magix-601 spectrometer. NH_3 temperature-programmed desorption (NH_3-TPD) was carried out with an Autochem 2920 equipment (Micromeritics). In the analysis, all SAPO-34 zeolite samples were activated at 873 K for 1 h under He atmosphere, and cooled down and saturated with NH_3 at 373 K for 0.5 h. Then, the samples were purged with He for 0.5 h, followed by NH_3 desorption from 373 to 873 K under He atmosphere. The chemical compositions of SAPO-34-5, SAPO-34-12, SAPO-34-12_HighSi, SAPO-34-17 and SAPO-34-50 samples measured by EDX are $Al_{0.422\pm0.000}P_{0.482\pm0.001}Si_{0.096\pm0.001}$, $Al_{0.439\pm0.033}P_{0.455\pm0.021}Si_{0.091\pm0.003}$, $Al_{0.426\pm0.019}P_{0.401\pm0.011}Si_{0.170\pm0.005}$, $Al_{0.427\pm0.003}P_{0.470\pm0.004}Si_{0.103\pm0.003}$ and $Al_{0.432\pm0.002}P_{0.457\pm0.005}Si_{0.110\pm0.006}$, respectively. The maximum deviations of silica content for all SAPO-34 zeolite samples were controlled

within 6%. The chemical compositions of SAPO-34-5, SAPO-34-12, SAPO-34-12_HighSi, SAPO-34-17 and SAPO-34-50 samples measured by XRF is $Al_{0.415}P_{0.488}Si_{0.097}$, $Al_{0.397}P_{0.511}Si_{0.093}$, $Al_{0.479}P_{0.351}Si_{0.170}$, $Al_{0.418}P_{0.490}Si_{0.092}$ and $Al_{0.434}P_{0.461}Si_{0.105}$, respectively. The NH_3-TPD profiles are plotted as the desorption 'rate' versus temperature and shown in Supplementary Figure 3. Peak deconvolution analysis of NH_3-TPD profiles was conducted by Gaussian- Lorentzian function. The quantities of medium and strong acidity of SAPO-34-5 (peak at 609 and 701 K), SAPO-34-12 (peak at 608 and 703 K), SAPO-12_HighSi (peak at 619 and 724 K), SAPO-34-17 (peak at 593 and 683 K), and SAPO-34-50 (peak at 613 and 707 K) samples are 1.01 ± 0.01, 1.09 ± 0.02, 1.72 ± 0.02, 1.05 ± 0.01 and 1.02 ± 0.02 $mmol \cdot g_{zeo.}^{-1}$, respectively. Relatively narrow distribution of silica content of individual SAPO-34 zeolite crystals is found for all samples (Supplementary Table 1). Comparison of silica contents measured by XRF and EDX reflects that the compositions at crystal surface and bulk crystals are similar. Note that a silica content of 0.1 corresponds to 1 $mmol \cdot g_{zeo.}^{-1}$ acid sites (Supplementary Table 1), the quantity of acid sites for individual crystal was estimated. The influences of distributions of quantity of acid sites on simulation results were examined.

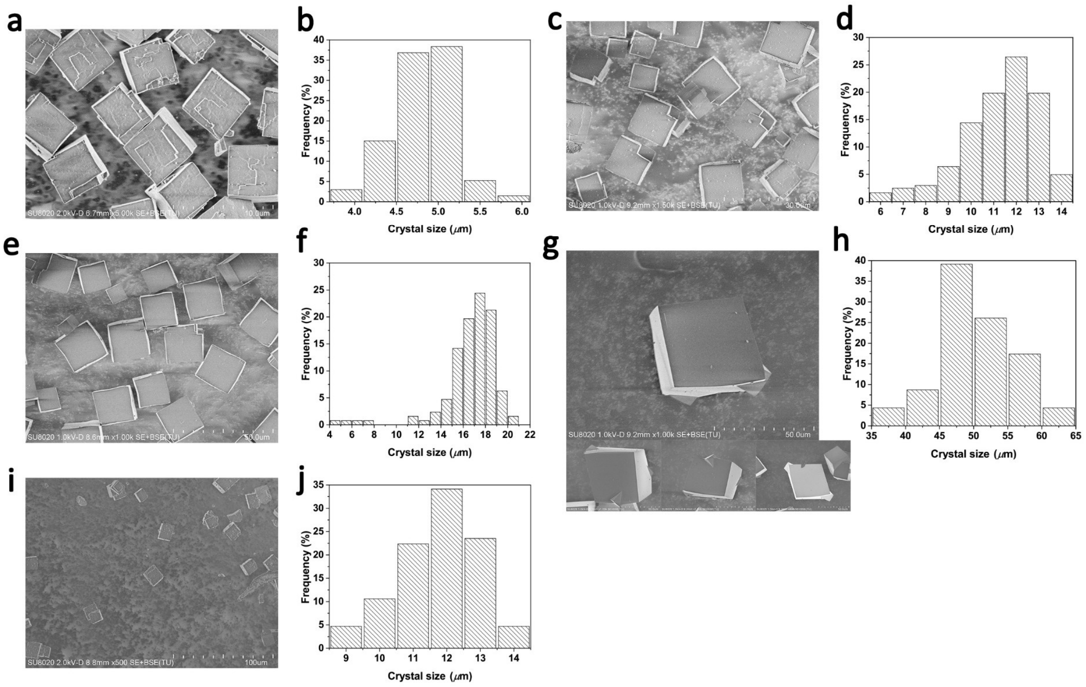

Supplementary Figure 2: Representative FE-SEM images of **a** SAPO-34-5, **c** SAPO-34-12, **e** SAPO-34-17, **g** SAPO-34-50 and **i** SAPO-34-12_HighSi samples. Crystal size distribution of **b** SAPO-34-5, **d** SAPO-34-12, **f** SAPO-34-17, **h** SAPO-34-50 and **j** SAPO-34-12_HighSi samples based on the statistics over 80-120 individual crystals.

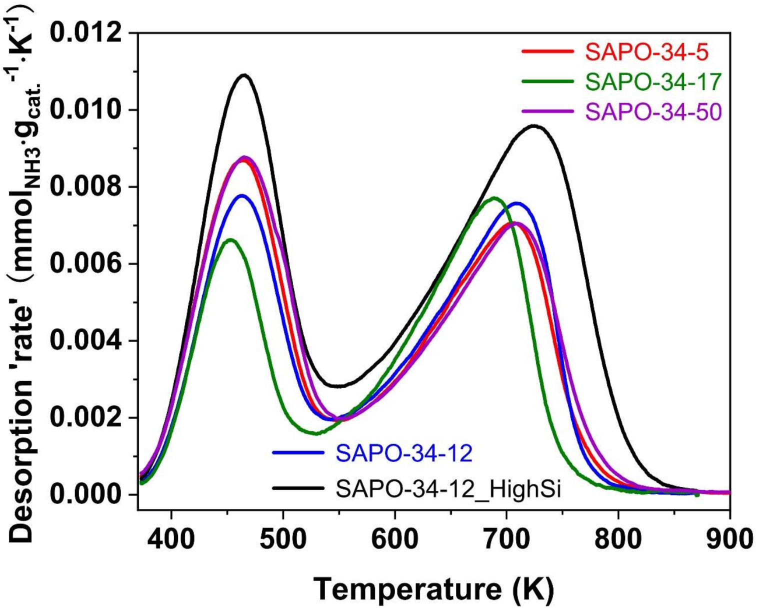

Supplementary Figure 3: NH_3-TPD profiles of SAPO-34-5, SAPO-34-12, SAPO-34-12_HighSi, SAPO-34-17 and SAPO-34-50 samples, observed during temperature ramp 10 K·min^{-1} following saturation with NH_3 at 373 K.

Supplementary Table 1: Statistical results of crystal size and silica content of individual SAPO-34 zeolite crystals and average quantity of acid sites and silica content of bulk SAPO-34 zeolite samples.

Samples	Crystal size (μm)	Silica content by EDX	Silica content by XRF	Bulk acidity by NH_3-TPD ($mmol\cdot g_{zeo.}^{-1}$)
SAPO-34-5	4.82 ± 0.36	$Si_{0.096 \pm 0.001}$	$Si_{0.097}$	1.01 ± 0.01
SAPO-34-12	11.17 ± 1.80	$Si_{0.091 \pm 0.003}$	$Si_{0.093}$	1.09 ± 0.02
SAPO-34-12_HighSi	11.68 ± 1.22	$Si_{0.170 \pm 0.005}$	$Si_{0.170}$	1.72 ± 0.02
SAPO-34-17	16.92 ± 1.66	$Si_{0.103 \pm 0.003}$	$Si_{0.092}$	1.05 ± 0.01
SAPO-34-50	47.08 ± 3.50	$Si_{0.110 \pm 0.006}$	$Si_{0.105}$	1.02 ± 0.02

The N_2 adsorption/desorption was conducted with a Micrometritics ASAP 2020 instrument at 77 K after the sample was degassed at 623 K under vacuum for 6 h. In Supplementary Figure 4, typical type I isotherms for microporous materials are observed for all SAPO-34 zeolite samples. The results of textural properties of SAPO-34 zeolites are summarized in Supplementary Table 2.

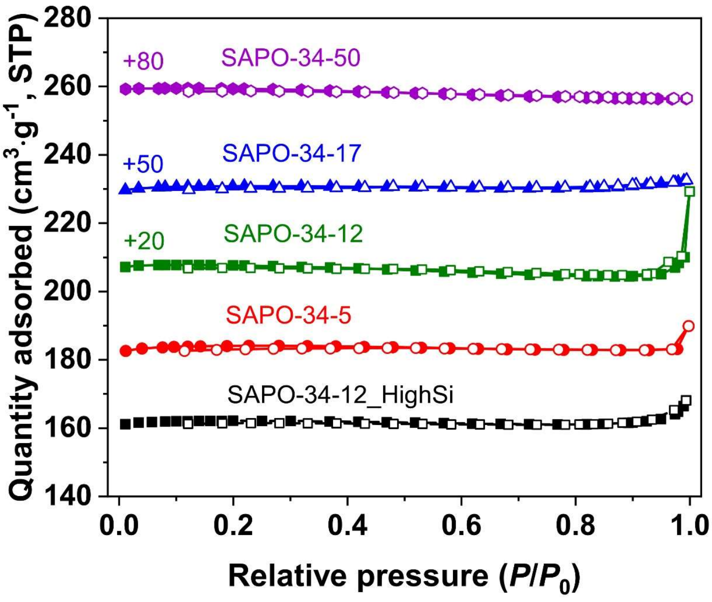

Supplementary Figure 4: N_2 adsorption and desorption isotherms at 77 K of SAPO-34-5, SAPO-34-12, SAPO-34-12_HighSi, SAPO-34-17 and SAPO-34-50 zeolite samples.

Supplementary Table 2: Textural properties of SAPO-34-5, SAPO-34-12, SAPO-34-12_HighSi, SAPO-34-17 and SAPO-34-50 zeolite samples by nitrogen adsorption and desorption isotherms at 77 K.

Sample	Surface area ($m^2 \cdot g_{zeo.}^{-1}$)		Pore volume ($cm^3 \cdot g_{zeo.}^{-1}$)	
	S_{total}[a]	S_{micro}[b]	V_{total}[c]	V_{micro}[b]
SAPO-34-5	552	549	0.28	0.28
SAPO-34-12	579	574	0.30	0.29
SAPO-34-12_HighSi	514	511	0.25	0.25
SAPO-34-17	556	552	0.28	0.28
SAPO-34-50	522	516	0.26	0.26

[a] BET surface area determined from multipoint method. [b] S_{micro} (micropore area) and V_{micro} (micropore volume) determined from the t-plot method. [c] V_{total} (total volume) is determined from adsorbed volume at $P/P_0 = 0.98$.

Supplementary Note 2: Experiments and reaction-diffusion simulations of MTO reaction

The methanol conversion and product selectivity in experiments of MTO reaction are shown in Supplementary Figure 5. The long-chain hydrocarbons such as C_4, C_5 and C_6 are lumped to C_{4+}, and paraffins including methane, ethane and propane are lumped to alkanes. In Supplementary Figure 5, at high temperature, the induction period is hardly observed due to high reactivity of methanol[2]. At the initial stage, the olefins-based cycle is dominant. The selectivity of ethylene is low, and propane and C_{4+} are relatively abundant at this stage. With MTO reaction proceeding, the selectivity of C_{4+} and propylene demonstrates a continuous decrease, meanwhile and the amount of carbonaceous species show a rapid increase (see Figure 6e). This indicates a shift of the predominant mechanism from olefins-based cycle to aromatics-based cycle[3], and correspondingly the selectivity of ethylene is increased. After that, significant decrease of methanol conversion can be observed, and the deactivation stage starts, which is featured by dramatical decrease of the selectivity of propylene and C_{4+} and notable increase of the selectivity of alkanes. This is due to the rapid formation of coke precursors within SAPO-34 zeolite crystal, which on the other hand promotes the hydrogen transfer, and on the other hand significantly hinders the diffusion of gas molecules (see Supplementary Figure 16). By comparing the MTO reaction over SAPO-34-5, SAPO-34-12, SAPO-34-17 and SAPO-34-50 samples, we found that zeolite crystals with smaller size show a prolonged catalyst lifetime.

In this work, we also show the results of reaction-diffusion simulations of MTO

reaction catalyzed by SAPO-34 zeolites at catalyst ensemble scale, focusing on the catalytic lifetime and selectivity of products.

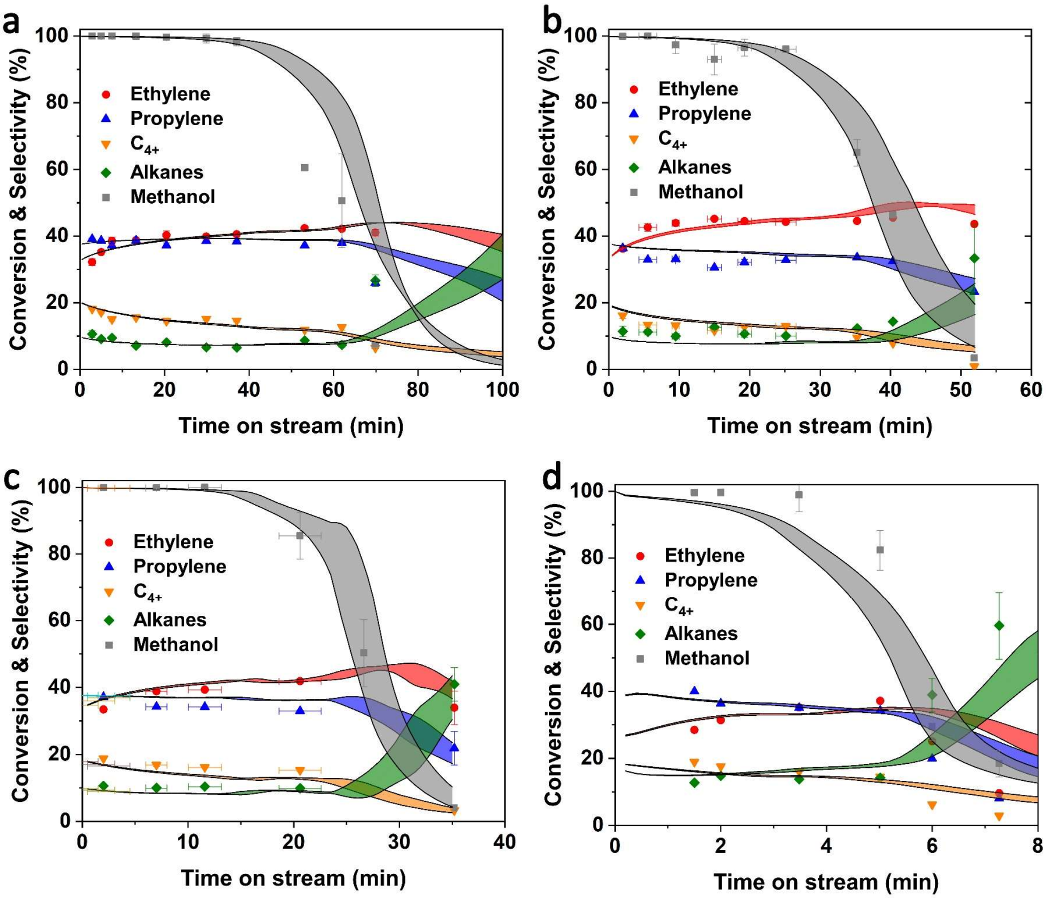

Supplementary Figure 5: Methanol conversion and product (ethylene, propylene, C_{4+} and alkanes) distribution as a function of TOS during MTO reaction in **a** SAPO-34-5 (4.82 ± 0.36 μm), **b** SAPO-34-12 (11.17 ± 1.80 μm), **e** SAPO-34-17 (16.92 ± 1.66 μm), **f** SAPO-34-50 (47.08 ± 3.50 μm) samples. The quantity of acid sites is 1.00 ± 0.06 mmol·$g_{zeo.}^{-1}$. Solid lines represent the simulated results and discrete points represent the experimental data. The error band is standard error of simulation results. Experimental conditions: T = 723 K, $WHSV$ = 5.0 ± 0.1 g_{MeOH}·$g_{zeo.}^{-1}$·h^{-1}, partial pressure of methanol of 0.28 bar.

Based on the *in-house* developed reaction-diffusion simulation code, the MTO reactions over SAPO-34-5, SAPO-34-12, SAPO-34-17 and SAPO-34-50 samples at 723 K and $WHSV$ = 5.0 ± 0.1 g_{MeOH}·$g_{zeo.}^{-1}$·h^{-1} were simulated[4, 5]. The change of loading of component i with time on stream (TOS) due to the reaction and diffusion is described

by the partial differential equations

$$\frac{\partial q_i}{\partial t} = -\nabla \cdot \overrightarrow{\mathbf{N}_i} + r_i, \qquad \text{(Supplementary Equation 1)}$$

where q_i is the mole loading of component i inside crystal, $\overrightarrow{N_i}$ the molecular flux of component i, r_i the reaction rate of component i, and t the TOS. The flux $\overrightarrow{N_i}$ can be predicted by Maxwell-Stefan diffusion theory. For n-component diffusion we have

$$-\frac{\theta_i}{RT}\nabla\mu_i = \sum_{\substack{j=1\\ j\neq i}}^{n} \frac{q_j\overrightarrow{\mathbf{N}_i} - q_i\overrightarrow{\mathbf{N}_j}}{q_i^{\mathrm{sat}} q_j^{\mathrm{sat}} D_{ij}} + \frac{\overrightarrow{\mathbf{N}_i}}{q_i^{\mathrm{sat}} D_i}, \qquad \text{(Supplementary Equation 2)}$$

where μ_i is the molar chemical potential of component i, R the ideal gas constant, T the absolute temperature, q_i^{sat} the saturation loading of component i, D_i the Maxwell-Stefan intracrystalline diffusivity of component i, D_{ij} the exchange diffusivity between component i and j, and θ_i the fractional occupancies of component i, which is defined as $\theta_i = q_i/q_i^{\mathrm{sat}}$. The gradient of the chemical potential can be expressed by

$$\frac{q_i}{RT}\nabla\mu_i = \sum_{j=1}^{n} \Gamma_{ij}\nabla q_j, \Gamma_{ij} = \frac{q_i}{f_i}\frac{\partial f_i}{\partial q_j}, \qquad \text{(Supplementary Equation 3)}$$

where f_i is the partial fugacity of component i. The relation between f_i and q_i can be obtained based on the ideal adsorbed solution theory (IAST). Thus, the Supplementary Equation 2 can be further written in matrix form as

$$(\overrightarrow{\mathbf{N}}) = -[B]^{-1}[\Gamma]\nabla(q), \qquad \text{(Supplementary Equation 4)}$$

where the elements of matrix $[B]$ is

$$B_{ii} = \frac{1}{D_i} + \sum_{\substack{j=1\\ j\neq i}}^{n} \frac{\theta_j}{D_{ij}}, B_{ij(i\neq j)} = -\frac{q_i^{\mathrm{sat}}}{q_j^{\mathrm{sat}}}\frac{\theta_i}{D_{ij}}. \qquad \text{(Supplementary Equation 5)}$$

The boundary condition of catalyst crystal is

$$q_i \big|_{a/2} = y\left(f_i^{\mathrm{gas}}\right). \qquad \text{(Supplementary Equation 6)}$$

where a is the crystal size of zeolite and function y represents the adsorption at the

external surface from gas phase.

By use of Supplementary Equations 1, 4 and 6, the reaction-diffusion process inside a zeolite crystal can be described. The matrix of thermodynamic correlation factors [Γ] in Supplementary Equation 4 can be calculated by IAST, based on the parameters of the pure-component isothermal adsorption equilibrium. The exchange diffusivity D_{ij} can be related to the intracrystalline diffusivity of component i by the interpolation formula

$$q_i^{\mathrm{sat}} D_{ij} = \left[q_j^{\mathrm{sat}} D_i\right]^{\frac{q_i}{q_i+q_j}} \left[q_i^{\mathrm{sat}} D_j\right]^{\frac{q_j}{q_i+q_j}} = q_j^{\mathrm{sat}} D_{ji}. \qquad \text{(Supplementary Equation 7)}$$

The intracrystalline diffusivity D_i and adsorption isothermals of pure component i can be obtained by molecular dynamic (MD) simulations. For the molecular diffusion inside SAPO-34 zeolites, the transport diffusivity and self-diffusivity of methane, methanol, ethylene, propylene, *n*-butylene were measured at the low temperature (293-333 K)[6, 7]. The diffusivities are extrapolated to high temperature by diffusion activation energy, which are also verified by MD simulations. Similarly, the adsorption isotherms of these components were measured at the low temperature[6]. During MTO reaction, the effect of retained hydrocarbons on adsorption is quantified by

$$q_i^{\mathrm{sat,coke}} = q_i^{\mathrm{sat}} \left(\frac{q_{\mathrm{coke,max}} - q_{\mathrm{coke}}}{q_{\mathrm{coke,max}}}\right)^2 \qquad \text{(Supplementary Equation 8)}$$

where q_i^{sat} is the saturated adsorption capacity of component i ($\mathrm{kmol \cdot kg_{zeo.}^{-1}}$), q_{coke} the amount of retained carbonaceous species in SAPO-34 zeolites at different TOS (wt %) and $q_{\mathrm{coke}}^{\mathrm{max}}$ the amount of retained carbonaceous species after deactivation (wt %). In our kinetic model, for simplicity, we defined a virtual HCP species that is a lump of active carbonaceous species covering the acid sites. In this way, we assume that 1 mol

HCP species would cover 1 mol acid site, and use a virtual molecular weight of acid site of 140 $g \cdot mol^{-1}$, i.e. the average molecular weight of HCP species, in the simulations. The virtual molecular weight, despite being easily implemented in the model, is overestimated. But in the simulations, the acid sites do not appear in the mass balance of hydrocarbon conversions, and the virtual molecular weight of acid sites will not affect the simulation results of the quantity of acid sites for an individual SAPO-34 zeolite crystal. In our simulations, we obtained that the quantity of acid sites for an individual SAPO-34 zeolite crystal is about 1.00 ± 0.06 $mmol \cdot g_{zeo.}^{-1}$.

The effect of carbonaceous species on the intracrystalline diffusivity of gas molecules can be related to the formation of HCP species and coke precursors

$$D_i^{\mathrm{coke}} = D_i \exp(-A_i q_{\mathrm{HCPs}}) \exp(-B_i q_{\mathrm{coke}}) \quad \text{(Supplementary Equation 9)}$$

where A_i and B_i ($kg_{zeo.} \cdot kg^{-1}$) are, respectively, the dimensionless quantity of HCP species an coke precursor deposited in SAPO-34 zeolites. Here q_{HCPs} and q_{coke} are the mass loading of HCP species and coke precursor inside catalyst ($kg \cdot kg^{-1}$).

It is well-known the dual-cycle mechanism can better describe the MTO reaction[3]. In the Supplementary Table 3, the reaction network and corresponding kinetic constants are listed, which were obtained by fitting experimental data with SAPO-34-5, SAPO-34-12, SAPO-34-17 and SAPO-34-50 samples, respectively, as shown in Supplementary Figure 5 and Fig.6 a and b. Note that it is hard, if not impossible, to directly verify the kinetic constants, we have examined the rationality of kinetic constants against the results of density functional theory (DFT) calculations[8, 9, 10]. DFT calculations show that the propylene is more likely to be formed than ethylene during

olefins-cycle[8, 9], and we indeed found that the reaction kinetic constant of propylene formation is larger than that of ethylene formation in the olefins-cycle. We obtained that the kinetic constant of ethylene formation is larger than that of propylene formation in aromatics-cycle, which are well consistent with the DFT calculations[10] that show that the overall free energy of ethylene formation is lower than that of propylene formation. MTO reaction catalyzed by SAPO-34-12_HighSi sample as extended study case further validates the applicability of kinetic constants as shown in Supplementary Figure 6.

The catalyst bed is considered to be a fixed-bed reactor composed of a number of perfectly mixed sub-reactors in series

$$\chi \frac{\partial \rho_{i,k}}{\partial t} = k_f\left(\rho_{i,k-1} - \rho_{i,k}\right) + \frac{3(1-\chi)}{a/2} n_i^{\mathrm{surf}}. \quad \text{(Supplementary Equation 10)}$$

where χ (dimensionless) is the voidage of catalyst bed (χ = 0.5), k_f the ratio of volume flow rate of feed to gas phase volume (s^{-1}), and $\rho_{i,k-1}$ the density of compound i in the (k^{-1}) th sub-reactor and n_i the net mass flux of compound i at boundary surface of zeolite crystal ($kg \cdot m^{-2} \cdot s^{-1}$).

Supplementary Table 3: Detailed reaction network and corresponding kinetic constants in this study.

No.	Reaction type	Reaction	Kinetic constant ($m^3 \cdot kg^{-1} \cdot s^{-1}$)
1	Olefin-base	MeOH + BAS → $C_2^=$ + H_2O	4.85 ± 0.15
2		MeOH + BAS → $C_3^=$ + H_2O	5.40 ± 0.10
3		MeOH + BAS → C_{4+} + H_2O	3.10 ± 0.01
4		MeOH + BAS → Alkanes + H_2O	1.23 ± 0.20
5	HCP species formation	$C_3^=$ + BAS → HCPs	0.04 ± 0.00
6		C_{4+} + BAS → HCPs	0.10 ± 0.00
7	Aromatic-base	MeOH + HCPs → C_2 + H_2O	6.00 ± 0.71

8		MeOH + HCPs → $C_3^=$ + H_2O	3.35 ± 0.82
9	Coke precursors formation	MeOH + HCPs → Coke + H_2O	0.07 ± 0.01
10		$C_2^=$ + HCPs → Coke	0.02 ± 0.00
11		$C_3^=$ + HCPs → Coke	0.06 ± 0.01
12		C_{4+} + HCPs → Coke	0.04 ± 0.01
13	Hydrogen transfer reaction	MeOH + Coke → Alkanes + H_2O	0.06 ± 0.00
14		$C_2^=$ + Coke → Alkanes	0.10 ± 0.03
15		$C_3^=$ + Coke → Alkanes	0.02 ± 0.00

BAS: Brønsted acidity; HCPs: Hydrocarbon pool species; Coke: coke precursors.

The systematic errors of simulations caused by crystal size distribution (within 15%), acid sites distribution (within 6%) and error of *WHSV* (within 2%) were evaluated as shown in Supplementary Table 4.

Supplementary Table 4. Average standard error of simulations of methanol conversion, selectivity of ethylene, propylene, C_{4+} and alkanes, relative quantity of retained acidity and coke content caused by error of crystal size, acidity and *WHSV*.

Error margin	Conversion	Sel. $C_2^=$	Sel. $C_3^=$	Sel. $C_4^=$	Sel. Alk.	Acidity	Coke
± 10% crystal size	2.82	0.19	0.27	0.27	0.39	1.22	0.23
± 16% crystal size	5.32	0.87	1.57	0.64	2.60	1.90	0.36
± 10% acidity	0.99	0.13	0.15	0.20	0.27	1.59	0.32
± 10% *WHSV*	3.13	0.14	0.21	0.20	0.33	1.12	0.22

We synthesized a SAPO-34 zeolite sample with high silica content to test the influence of silica content on MTO reaction by experiments. This sample, namely SAPO-34-12_HighSi, has average crystal size of 11.68 ± 1.22 μm, chemical composition of $Al_{0.426\pm0.019}P_{0.401\pm0.011}Si_{0.170\pm0.005}$ and quantity of acid sites of 1.72 ± 0.02

mmol·$g_{zeo.}^{-1}$. Relatively uniform distributions of crystal size and silica content of SAPO-34-12_HighSi sample are also granted (Supplementary Table 1). Based on the estimated parameters, the MTO reaction catalyzed by SAPO-34-12_HighSi was simulated. The simulated results, as shown in Supplementary Figure 6, manifest that increasing silica content of SAPO-34 zeolites, i.e. quantity of acid sites, will shorten catalyst lifetime, decrease initial selectivity of ethylene, and increase initial selectivity of C_{4+}, which agree well with the experimental results. Compared with results of SAPO-34-12, higher relative quantity of acid sites (59.54%) and less quantity of carbonaceous species (16.75 wt%) retained in deactivated SAPO-34-12_HighSi sample can be observed. To understand MTO reaction catalyzed by SAPO-34-12_High Si, the simulated spatiotemporal evolution of molecules and acid sites during MTO reaction are shown in Supplementary Figure 6e and Supplementary Figure 7. In particular, the spatiotemporal evolution of HCP species and coke precursors inside SAPO-34-12_HighSi zeolite crystal during MTO reaction has been verified by SIM results as shown in Supplementary Figure 6d. As can be seen in Supplementary Figure 6a and e, more quantity of alkanes, HCP species and coke precursors are formed at the initial stage of MTO reaction over SAPO-34-12_HighSi than that over SAPO-34-12. This can be explained as that a higher quantity of acid sites would promote the occurrence of cyclization and hydrogen transfer reaction. We also observed from Supplementary Figure 6e that, at the initial stage, increasing the quantity of acid sites causes HCP species and coke precursors are accumulated at the region close to rim (~ 2 μm from the rim). Part of olefinic products generated at this region can diffuse toward the crystal

center and be rapidly converted to HCP species and then coke precursors. Therefore, the accumulation of coke precursors at the crystal center can be observed. Subsequently, at the crystal rim, high concentrated HCP species can rapidly react with methanol and olefinic products to further form coke precursors, and coke precursors formed at the rim significantly hinder the diffusion of reactant into crystal interior. In this connection, in the deactivated SAPO-34-12_HighSi, plenty of acid sites and HCP species trapped in the crystal are inaccessible for reactants, which can be found in Supplementary Figure 6b and c. By use of the multiscale reaction-diffusion simulations, the insights of insufficient utilization of acid sites and HCP species during MTO reaction catalyzed by SAPO-34 with high silica content are unveiled.

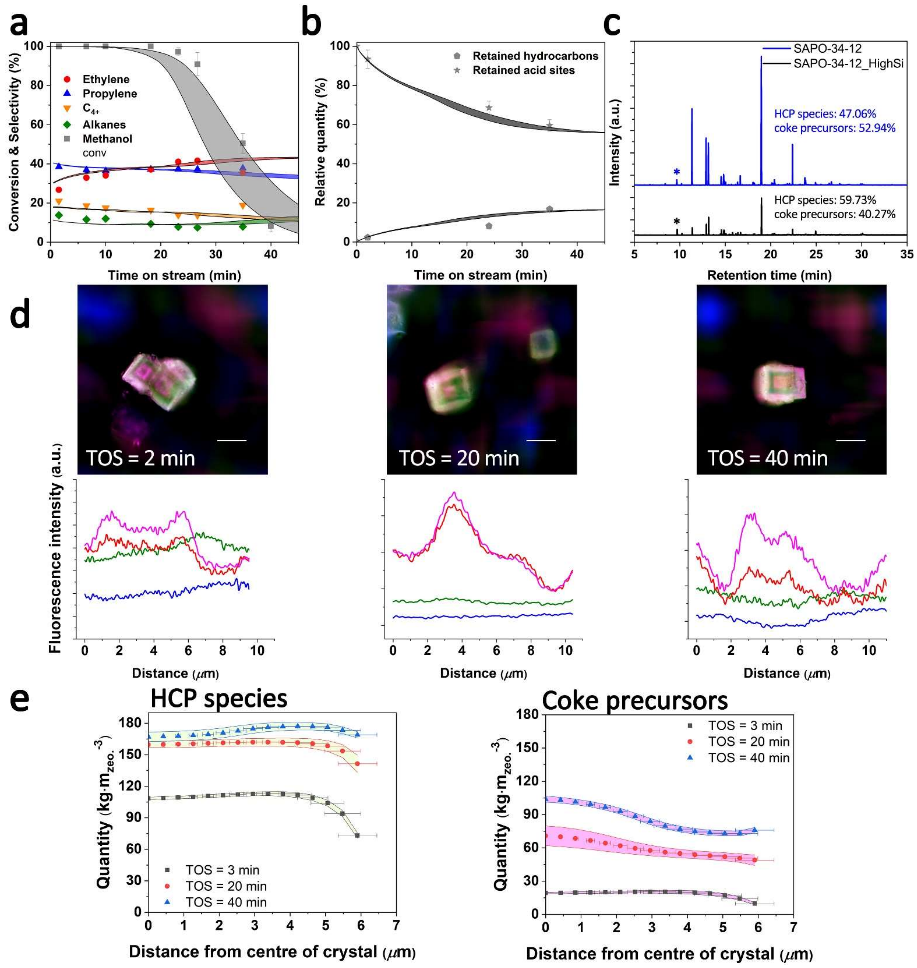

Supplementary Figure 6: Comparison of MTO reactions catalyzed by SAPO-34-12_HighSi sample (11.68 ± 1.22 μm) between simulated and experimental results. **a** Conversion of methanol and selectivity of gas products. **b** Evolution of quantity of retained acid sites and carbonaceous species in bulk SAPO-34 zeolites. **c** Component analysis of carbonaceous species retained in SAPO-34-12 and SAPO-34-12_HighSi zeolite samples after catalytic deactivation by GC-MS. * represents the internal standard. **d** Spatiotemporal evolution of carbonaceous species inside crystal obtained by SIM. **e** Simulated results of spatiotemporal evolution of HCP species and coke precursors inside crystal. The quantity of acid sites is 1.70 ± 0.10 $mmol \cdot g_{zeo.}^{-1}$. The error band is standard error of simulation results. Experimental conditions: T = 723 K, $WHSV$ = 5.0 ± 0.1 $g_{MeOH} \cdot g_{zeo.}^{-1} \cdot h^{-1}$, partial pressure of methanol of 0.28 bar.

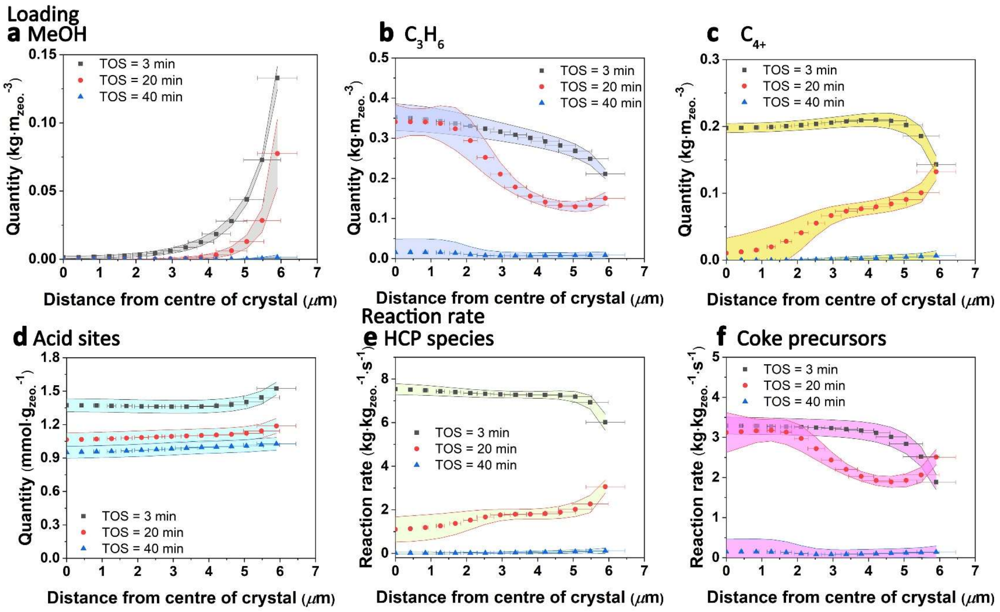

Supplementary Figure 7: Simulated results of loading of **a** methanol, **b** propylene, **c** C_{4+} and **d** acid sites and reaction rate of **e** HCP species and **f** coke precursors inside SAPO-34 zeolite crystal during MTO reactions catalyzed by SAPO-34-12_HighSi sample. The quantity of acid sites is 1.70 ± 0.10 mmol·$g_{zeo.}^{-1}$. *WHSV* is 5.0 ± 0.1 g_{MeOH}·$g_{zeo.}^{-1}$·h^{-1}. The error band is standard error of simulation results.

Supplementary Note 3: Characterization of acidity and retained carbonaceous species

The measured results of acidity by DRIFT were used as input for reaction-diffusion modelling at the catalyst ensemble scale as shown in Figure 1a. DRIFT spectra were recorded on a Bruker Tensor 27 instrument supplied with MCT detector[11]. The spent SAPO-34 zeolite was contained in a diffuse reflectance infrared cell with ZnSe window. The sample was heated at 623 K for 1 h under N_2 to remove adsorbed water inside the zeolites, then the absorbance spectra were obtained by collecting 32 scans at 4 cm^{-1}. In Supplementary Figure 8, DRIFT spectra show the detailed vibrational properties of the zeolitic framework and retained hydrocarbons. Two bands at around 3618 and 3596 cm^{-1} are assigned to the stretching vibration of Si(OH)Al (Brønsted acidity). Correspondingly, the relative quantity changes of Brønsted acidity during MTO reaction in different SAPO-34 zeolites are shown in Figure 6b. As shown in Figure 6b, after MTO deactivation, the smaller the crystal size, the less Brønsted acidity detected, which implies that the utilization of Brønsted acidity is more adequate in SAPO-34 zeolites with smaller crystal size. In the other words, it means that abundance of Brønsted acidity are retained inside SAPO-34-50 but inaccessible to gas molecules. The appearance of bands in the region 1700-1500 cm^{-1} suggests the formation of polycyclic aromatic hydrocarbons, which are associated with the stretching vibration of the C=C bond[12]. Similarly, the absorbance at 1620 cm^{-1} is relatively weak in SAPO-34-50, and the absorbance at 1620 cm^{-1} in SAPO-34 zeolites becomes stronger as the crystal size decreases. This indicates that increasing crystal size will lead to the prolonged diffusion

length, and thus the suppressed formation of polycyclic aromatic hydrocarbons. It can be explained by the inadequate accessibility of Brønsted acidity to gas molecules.

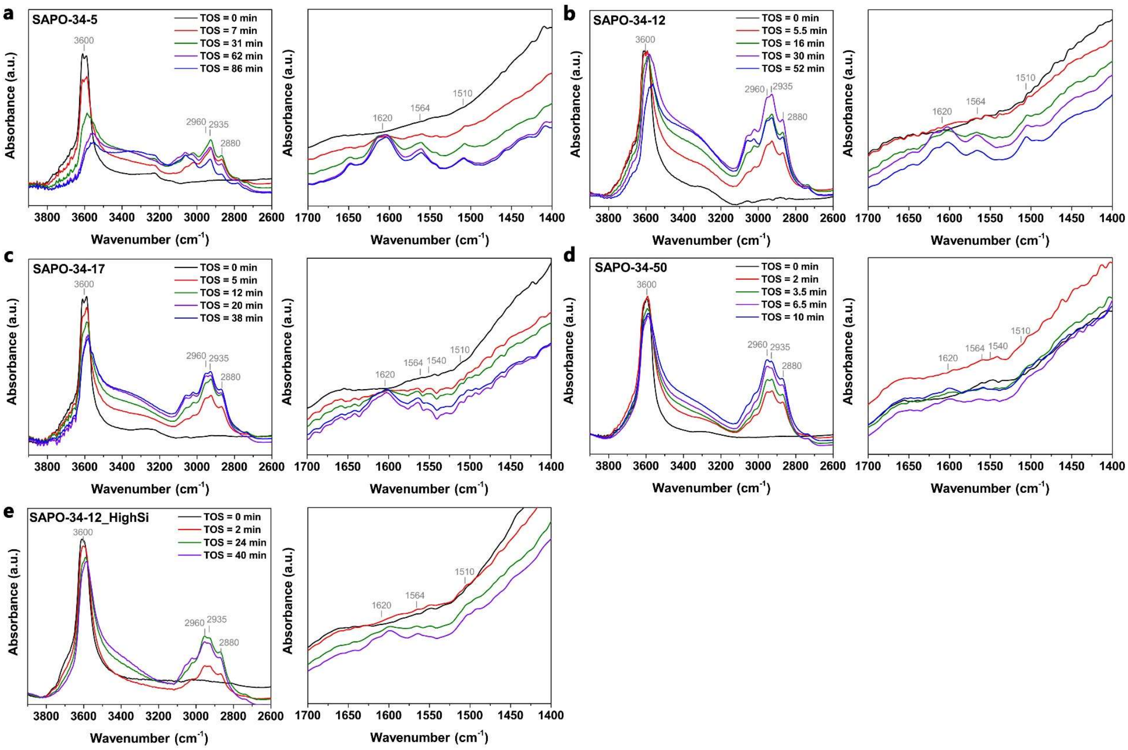

Supplementary Figure 8: DRIFT spectra on **a** SAPO-34-5, **b** SAPO-34-12, **c** SAPO-34-17, **d** SAPO-34-50 and **e** SAPO-34-12_HighSi zeolite samples during MTO reaction.

Thermogravimetric analysis (TGA) and differential thermogravimetry (DTG) were performed on SDT Q600. For each measurement, approximately 10 mg of spent SAPO-34 zeolites were heated under 100 mL·min^{-1} of air to 1173 K at a rate of 10 K·min^{-1}. With the assistance of TGA-DTG, H/C ratio of the retained carbonaceous species was first derived, which was then used to judge the degree of transformation of carbonaceous species. The derivative heat flow can reflect H/C ratio of retained carbonaceous species, e.g. carbonaceous species with lower H/C ratio would be burned to a relatively higher temperature $T_{G,max}$, which can be obtained from DTG profiles. As

shown in Supplementary Figure 9, $T_{G,max}$ for the SAPO-34-5 is 910 K, while there are two distinct $T_{G,max}$ for SAPO-34-50, i.e. 775 and 911 K. This suggests that, during MTO reaction, the retained carbonaceous species can be more readily transformed to coke precursor in zeolites with smaller crystal size.

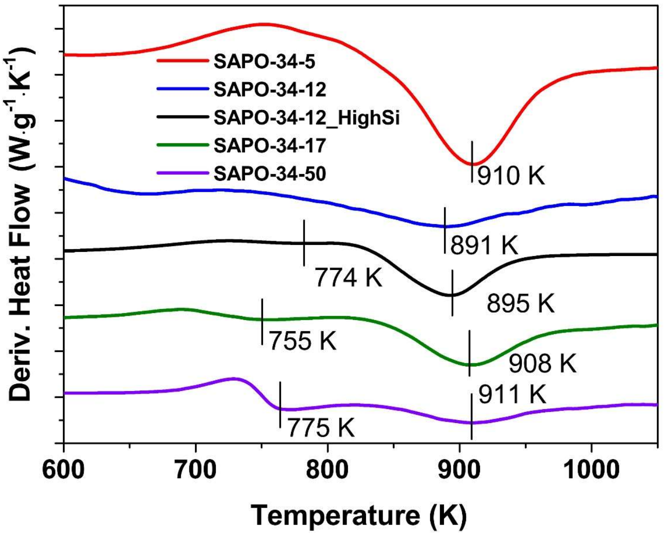

Supplementary Figure 9: DTG profiles of SAPO-34-5, SAPO-34-12, SAPO-34-12_HighSi, SAPO-34-17 and SAPO-34-50 zeolite samples at completed MTO deactivation stage.

Supplementary Note 4: Time-dependent density functional theory

In order to differentiate the carbonaceous species by structural illumination imaging (SIM) technique, we first performed time-dependent density functional theory (TDDFT) calculations to identify the excitation (first excitation energy, group state S_0) and emission (excited state S_1) wavelengths of different carbonaceous species. In doing so, all structure optimizations were carried without constraints at B3LYP/6-31G (d, p) levels, for both the ground and excited states of carbonaceous species in gas phase[13, 14]. The harmonic frequency calculations based on Gaussian 09 package[15] confirmed that the optimized structures correspond to the minimum-energy points with all the frequencies being positive. In the calculations, the confinement imposed by CHA topology was not considered owning to the very time-consuming computation. It is found that the deviation of excitation wavelength at confined state from that of gas phase carbonaceous species is about 30 nm[13]. The summaries of the calculated excitation and emission wavelengths of benzenic (B_n^+), naphthalenic (N_n^+), phenanthrenic (PH_n^+) and pyrenic (PYR_n^+) carbocations with *n* methyl substituents are shown in Supplementary Table 5. As can be seen, the excitation wavelengths of B_n^+, N_n^+, PH_n^+ and PYR_n^+ are situated around 390, 480, 560 and 640 nm, respectively, and the corresponding emission wavelengths are located in the range of 480-490, 500-520, 620-630 and 670-700 nm, respectively. Additionally, we performed TDDFT to estimate the phosphorescence behavior of carbonaceous species (state T_1). The phosphorescence signals of B_2^+, N_4^+, PH_0^+ and PYR_0^+ show a peak at 578, 568, 700 and 816 nm, respectively. Compared to that of fluorescence signal, the wavelength of

phosphorescence signal for given species is about 80 nm higher. It has been similarly observed by time resolved photoluminescence spectroscopy of the trimethyladamantylammonium hydroxide confined in **CHA** topology[16].

Supplementary Table 5: Wavelengths of excitation (first excitation energies, group state S_0)[13, 14] and emission (excited states S_1) of charged carbonaceous species in gas phase calculated at the B3LYP/6-31G (d, p) level of theory.

Species	Excitation wavelength (nm)	Excitation wavelength (nm) [a]	Oscillator strength of absorbance (-)	Emission wavelength (nm)	Oscillator strength of emission (-)
B_1^+	311	342	0.0799	482	0.0001
B_2^+	317	368	0.1058	484	0.0002
B_5^+	345	391	0.0751	492	0.0002
B_6^+	330	385	0.0778	499	0.0002
N_0^+	447	466	0.0024	503	0.0044
N_1^+	441	494	0.0003	496	0.0018
N_3^+	448	469	0.0027	503	0.0047
N_4^+	452	520	0.0002	508	0.0005
N_5^+	455	/	0.0014	515	0.0012
PH_0^+	550	559	0.0009	632	0.0021
PH_1^+	552	/	0.0009	623	0.0023
PYR_0^+	625	623	0.0005	672	0.0015
PYR_1^+	659	/	0.0007	665	0.0006

[a] Calculated results of TDDFT[13, 14], which were considered the confinement by CHA topology. B_n^+, N_n^+, PH_n^+ and PYR_n^+ stand for benzenic, naphthalenic, phenanthrenic and pyrenic carbocation with *n* methyl substituents, respectively.

Supplementary Note 5: Super resolution structured illumination microscopy

A unique feature of SIM fluorescence microscopy is its ability to detect the spatial location of retained carbonaceous species[17]. There are four channels with corresponding wavelengths in SIM, i.e. 405 (detection at 435-485 nm), 488 (detection at 500-545 nm), 561 (detection at 570-640 nm) and 640 nm (detection at 663-738 nm). These wavelengths can cover the characteristic area of excitation and emission wavelengths of B_n^+, N_n^+, PH_n^+ and PYR_n^+ as shown in Supplementary Table 5. In the measurements, each channel of SIM can work independently and the corresponding detector collects the emitted light with specified wavelengths. In MTO reactions over SAPO-34 zeolites, however, methylbenzenes B_n^+ and methylnaphthalene N_n^+ are shown to be activated carbonaceous species (i.e. HCP species)[18], while phenanthrene PH_n^+ and pyrene PYR_n^+ are considered to be coke precursors[18]. The identification of each single carbonaceous species via SIM, at current stage, is still quite challenging. As can be seen in Figure 2, however, it is feasible to differentiate the HCP species (B_n^+ and N_n^+) and coke precursors (PH_n^+ and PYR_n^+) inside SAPO-34 zeolites by SIM. In this work, we argue that since the wavelengths of illustration and detection of SIM are sufficiently close, the interference of phosphorescence could be neglected. For example, as in Supplementary Table 5, if the phenanthrenic carbocations PH_n^+ are excited at 561 nm, the fluorescence signal can be captured by the detector with working range of 570-640 nm while the phosphorescence signal with peak at ~ 700 nm can be hardly measured by the same detector. By use of SIM and advanced reconstructed algorithm, the super-resolution images can be computationally reconstructed[19, 20]. All images are

taken at the middle layer of crystal, and the locations of carbonaceous species are derived by distribution of fluorescence intensity along at the middle layer of SAPO-34 zeolite crystals.

The direct comparison between UV-vis spectra and SIM images is quite challenge. The absorbed and emissive response of a given carbonaceous species might be different even if it is excited at the same wavelength. Figure 2a shows the oscillator strength that represents the absorbance of carbonaceous species to excited wavelength, which manifests an overall decrease from B_n^+, N_n^+, PH_n^+ to PYR_n^+. On the meantime, in Figure 2b, the oscillator strength reflects the luminescent intensity of excited carbonaceous species shows an overall increase from B_n^+, N_n^+, PH_n^+ to PYR_n^+. These results indicate that the absorbance of a given carbonaceous species (e.g. PYR_n^+) to incident light at the wavelength of, for instance 640 nm, is weak while the emitted fluorescence signal excited by light at the same wavelength is much stronger. In this work, the fluorescence image obtained by SIM can qualitatively illustrate spatial distribution of a given type of carbonaceous species, i.e. HCP species and coke precursors, excited at a certain wavelength within an SAPO-34 zeolite crystal at certain reaction stage. However, due to the wavelengths of incident lights are different, the SIM images of one carbonaceous species cannot be directly compare to SIM images of another species. UV-vis spectra provide semiquantitative comparison of a given type of carbonaceous species excited at the same wavelength for different SAPO-34 zeolite samples at different MTO reaction stages. In this measurement, the temporal evolutions of HCP species and coke precursors inside SAPO-34 zeolite samples are mainly concerned. The quantities of

carbonaceous species were also obtained by thermogravimetric analysis (TGA) combined with dissolution/extraction experiments. The SIM and UV-vis spectra results are separately compared to the simulation work and partially validate the multi-scale reaction-diffusion model. The model is used to link the spatial and temporal evolution of carbonaceous species.

The spent SAPO-34 zeolite samples were obtained by the following steps. MTO reaction catalyzed by calcined SAPO-34 zeolites sample was performed in fixed-bed reactor, and, when MTO reaction reached specific methanol conversion, the methanol feed was switched to nitrogen flow until temperature of fixed-bed reactor was rapidly cooled down below 473 K. Then spent SAPO-34 zeolite sample was taken out and rapidly charged to the sealed tube, and stored in the desiccator. During the shift of spent SAPO-34 zeolite samples, the exposure time of samples to oxidising atmosphere, including storage time, was within 24 hours. SIM measurements under reactive conditions with a careful-designed experimental facility is highly desired for further study.

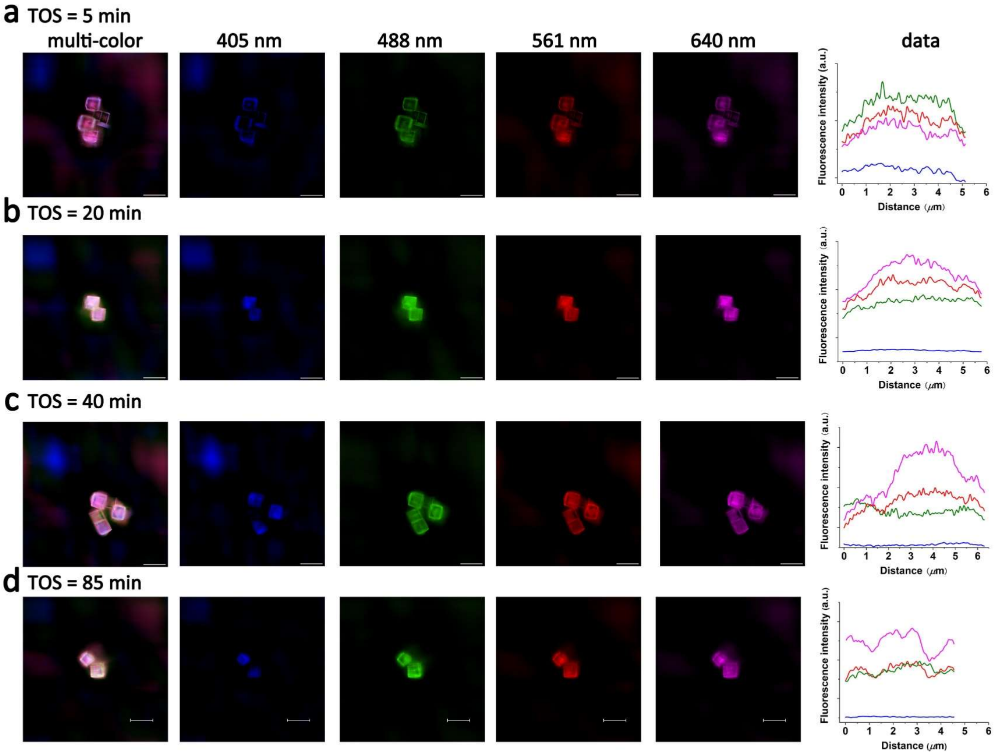

Supplementary Figure 10: SIM super-resolution images of the SAPO-34-5 zeolite crystals with TOS during the MTO reaction at 723 K. The false colors in the SIM images originate from the individual profile with a laser excitation of 405 nm (detection at 435~485nm, blue channel), 488 nm (detection at 500~545 nm, green channel), 561 nm (detection at 570~640 nm, red channel) and 640 nm (detection at 663~738 nm, pink channel). Intense fluorescence is detected in the central part of the crystal. The scale bar represents 10 μm.

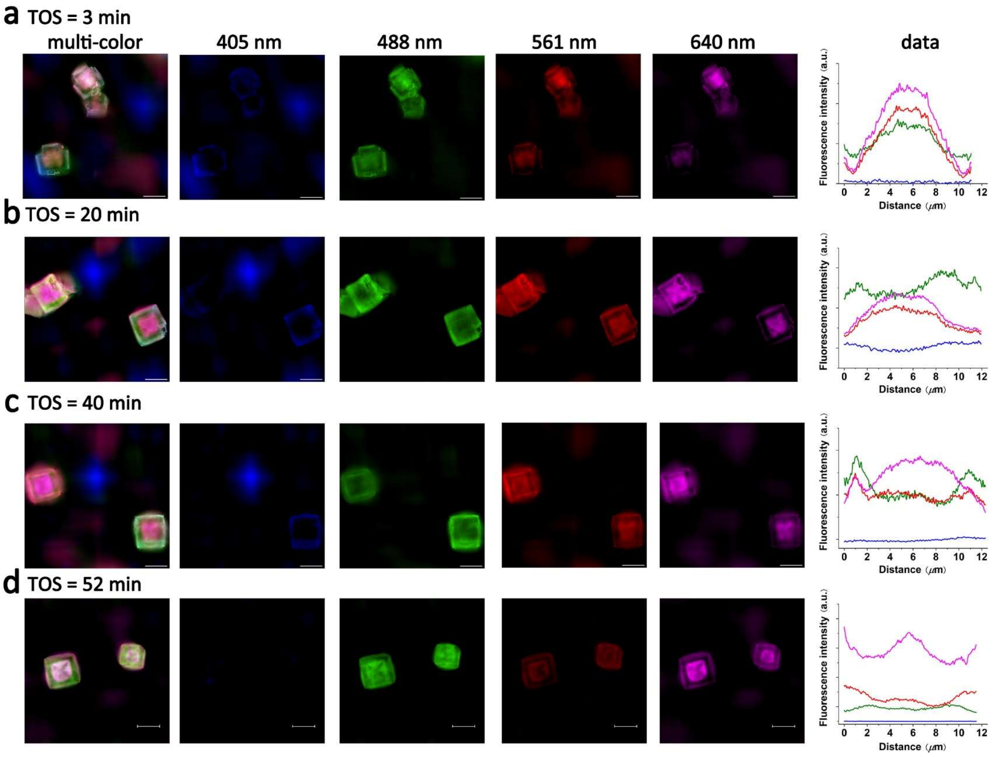

Supplementary Figure 11: SIM super-resolution images of the SAPO-34-12 zeolite crystals with TOS during the MTO reaction at 723 K. The false colors in the SIM images originate from the individual profile with a laser excitation of 405 nm (detection at 435~485nm, blue channel), 488 nm (detection at 500~545 nm, green channel), 561 nm (detection at 570~640 nm, red channel) and 640 nm (detection at 663~738 nm, pink channel). Intense fluorescence is detected in the central part of the crystal. The scale bar represents 10 μm.

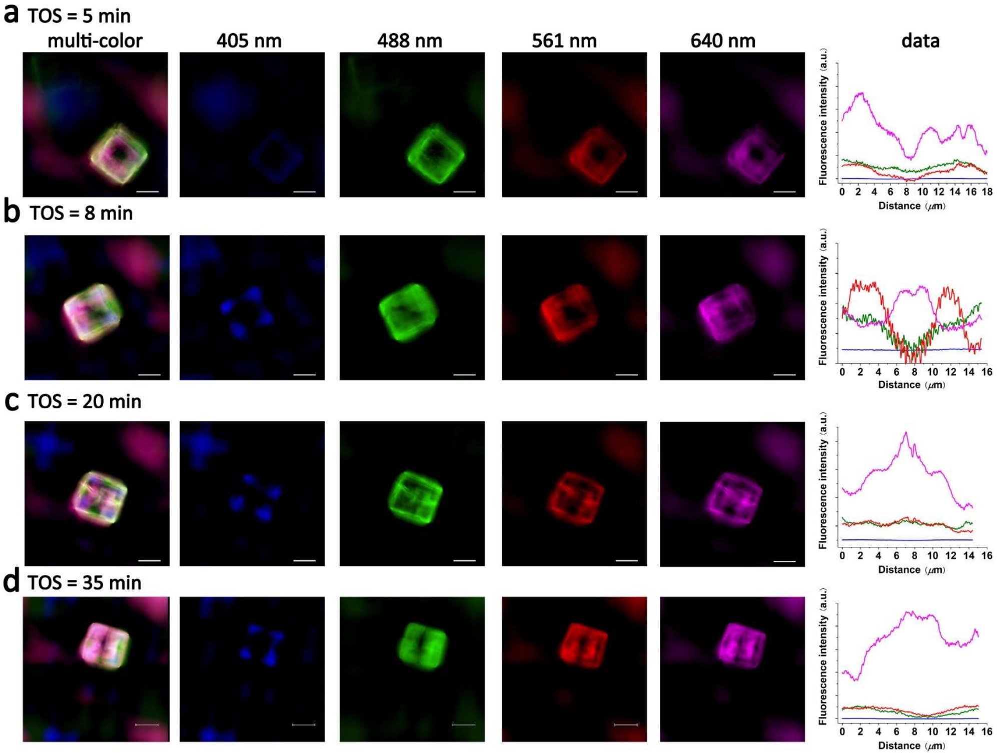

Supplementary Figure 12: SIM super-resolution images of the SAPO-34-17 zeolite crystals with TOS during the MTO reaction at 723 K. The false colors in the SIM images originate from the individual profile with a laser excitation of 405 nm (detection at 435~485nm, blue channel), 488 nm (detection at 500~545 nm, green channel), 561 nm (detection at 570~640 nm, red channel) and 640 nm (detection at 663~738 nm, pink channel). Intense fluorescence is detected in the central part of the crystal. The scale bar represents 10 μm.

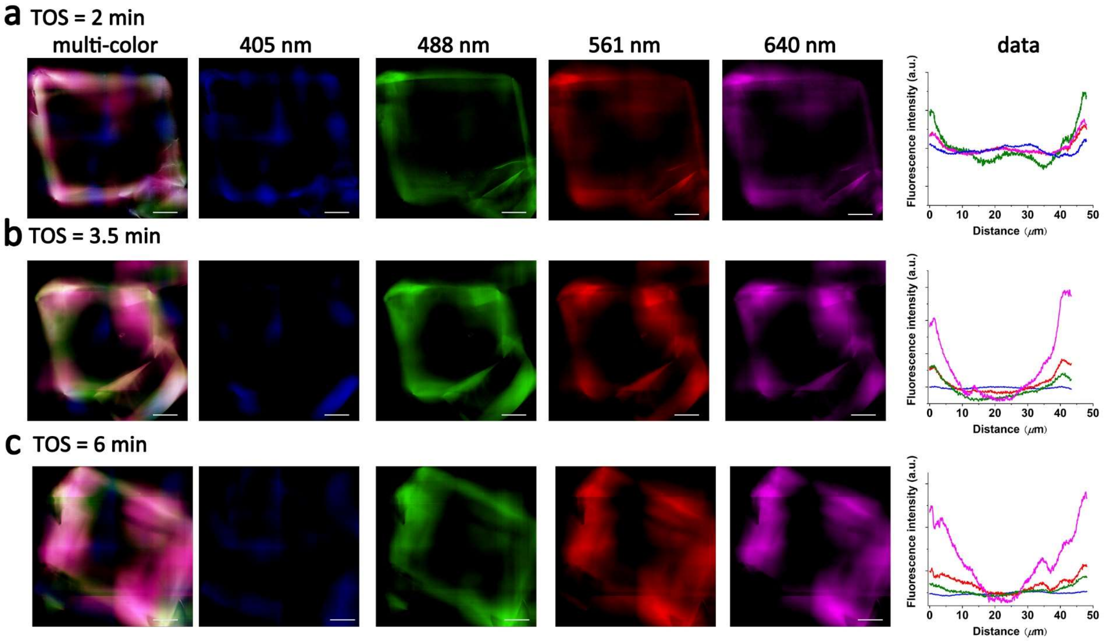

Supplementary Figure 13: SIM super-resolution images of the SAPO-34-50 zeolite crystals with TOS during the MTO reaction at 723 K. The false colors in the SIM images originate from the individual profile with a laser excitation of 405 nm (detection at 435~485nm, blue channel), 488 nm (detection at 500~545 nm, green channel), 561 nm (detection at 570~640 nm, red channel) and 640 nm (detection at 663~738 nm, pink channel). Intense fluorescence is detected in the central part of the crystal. The scale bar represents 10 μm.

Supplementary Note 6: Molecular dynamics simulations

The molecular diffusivities and adsorption isotherms were obtained from either molecular dynamics (MD) simulations or measured by uptake experiments over bulk SAPO-34 zeolite samples. As discussed in our previous work[6], for a narrow distribution of crystal size, the molecular diffusivities over bulk SAPO-34 zeolite samples can be used for individual zeolite crystal, which is verified by pulsed field gradient (PFG) NMR measurements. All MD simulations in this work were carried out using the Materials Studio simulation package (Accelrys Software). The adsorption of molecules in **CHA** structure were performed using the grand canonical Monte Carlo (GCMC) simulation method. Periodic boundary conditions were applied in all three directions. The interatomic interactions were described by the condensed-phase-optimized molecular potentials for atomistic simulation studies (COMPASS) force field. The Ewald & Group summation method has an Ewald accuracy of 10^{-5} kcal·mol^{-1} when used for calculating electrostatic potential energy. To achieve an equilibrium state, 10^7 Monte Carlo steps were carried out. The zeolitic framework with a rigid structure was considered. The metropolis scheme was used at a constant loading and constant temperature. To minimize the energy of constructed structures, all the structures were equilibrated by five anneal cycles from 250 to 750 K with a heating ramp of five to refine the conformation. Dynamics processes in the NVT ensemble, i.e. the number of particles (N), volume (V) and temperature (T) are keeping as constants, were performed for 6000 ps in 6000000 steps for methane, 15000 ps in 15000000 steps for methanol, 10000 ps in 10000000 steps for ethylene and 20000 steps in 20000000 steps for

propylene at 293 K and 723 K. The velocity Verlet algorithm was used to integrate the Newton's equations of motion with a time step of 1 fs. A cutoff radius of 18.5 Å was assumed for Lennard-Jones interaction potential calculation. The simulated temperature was controlled by a Nosé thermostat. The structures considered in this study are shown in Supplementary Figure 14. The mean square displacement (MSD) of methane, methanol, ethylene and propylene are shown in Supplementary Figure 15. The slope of MSD as a function of time was used to determine the self-diffusivity following Einstein relation[21]

$$MSD(\tau) = 2nD\tau + b \quad \text{(Supplementary Equation 11)}$$

where n is the dimension of framework (n = 1, 2 and 3 for 1D, 2D and 3D frameworks, respectively) and b the thermal factor arising from atomic vibrations.

To identify the effect of deposited carbonaceous species on intracrystalline diffusion of gas molecule, MD simulations were carried out. The detailed structures used in the simulation are shown in Supplementary Figure 14. First, to check the applicability of the used force field, MD simulations were conducted for methane and ethylene in $AlPO_4$-34 zeolite (**CHA** structure) under 298 K. As can be seen from Supplementary Figure 15a, the calculated intracrystalline self-diffusivities of methane ($D = 4.53 \times 10^{-10}$ $m^2 \cdot s^{-1}$) and ethylene ($D = 6.67 \times 10^{-11}$ $m^2 \cdot s^{-1}$) in $AlPO_4$-34 zeolite at 298 K are consistent with the experimental results of PFG NMR[7, 22]. Then the MD simulations of methane, ethylene, methanol and propylene in $AlPO_4$-34 zeolites loading with 0.5 per cage *p*-xylene, naphthalene and phenanthrene at 723 K were performed, respectively. As shown in Supplementary Figure 16, in the empty cage of $AlPO_4$-34 zeolite, self-

diffusivity increases in the order: propylene < ethylene ≈ methanol < methane. When the cage is loaded with *p*-xylene and naphthalene, the self-diffusivities of methane, methanol, ethylene and propylene slightly decrease (ca. D_{load}/D_{empty} = 0.3 ~ 1). When the cage is loaded with phenanthrene, the self-diffusivities of methane, methanol, ethylene and propylene drop markedly (ca. D_{load}/D_{empty} = 0.05 ~ 0.1). It can be concluded that the formation of aromatic hydrocarbons with more than three rings will dramatically limit molecular diffusion in **CHA** structure.

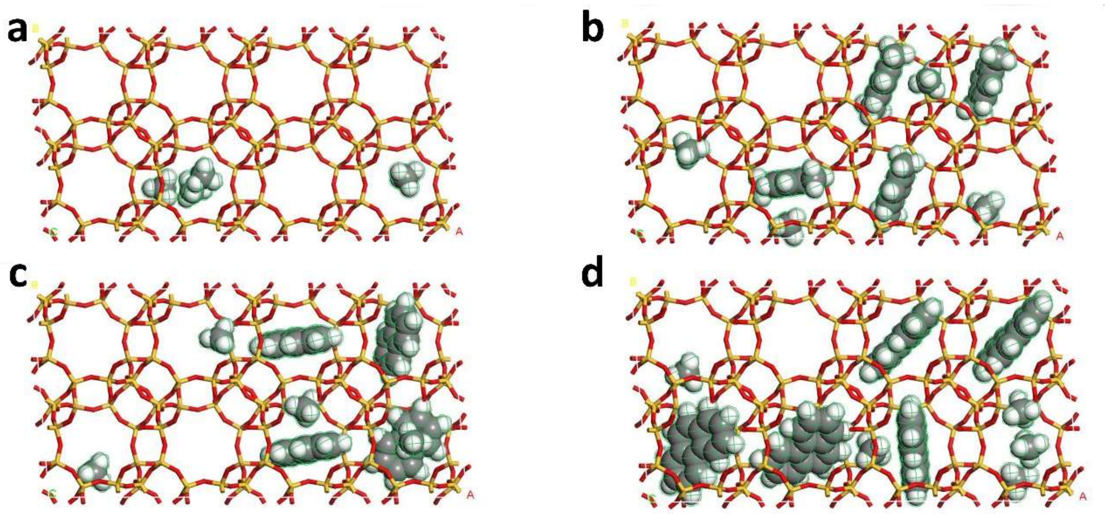

Supplementary Figure 14: Structure of $AlPO_4$-34 (**CHA**), loaded with 0.5 per cage methane molecules in cage with **a** empty, **b** 0.5 per cage *p*-xylene loading, **c** 0.5 per cage naphthalene loading and **d** 0.5 per cage phenanthrene loading. Atom colors: C (grey), H (white), O (red) and Al (yellow).

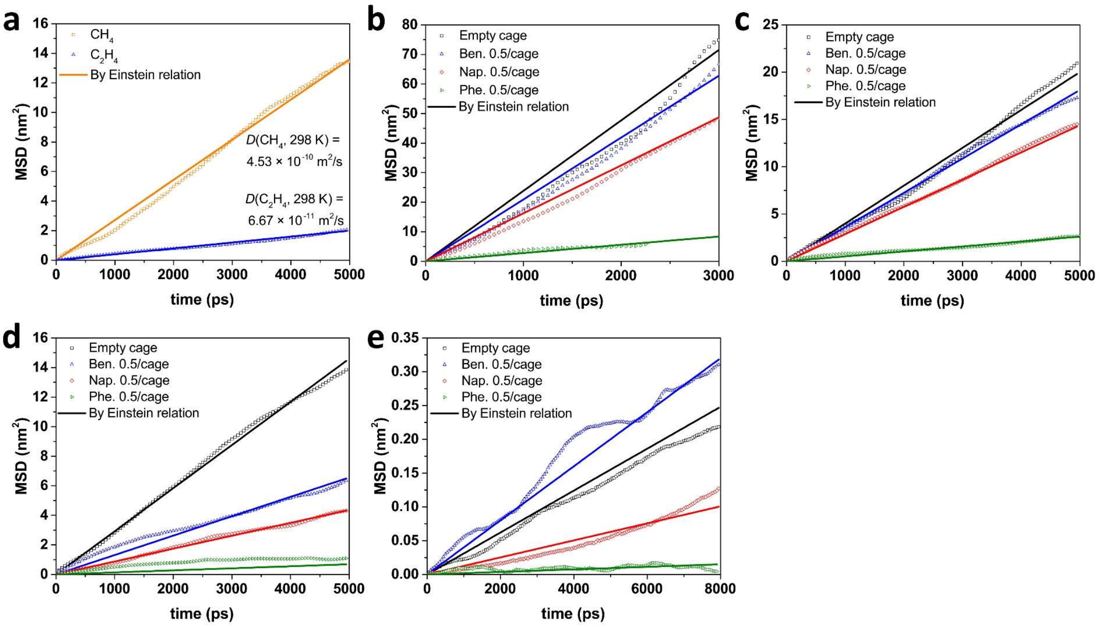

Supplementary Figure 15: **a** MSD of methane and ethylene diffusions in $AlPO_4$-34 zeolite and corresponding self-diffusivity at 293 K. MSD of **b** methane, **c** methanol, **d** ethylene and **e** propylene diffusion in $AlPO_4$-34 zeolite with empty, p-xylene loading, naphthalene loading and phenanthrene loading at 723 K.

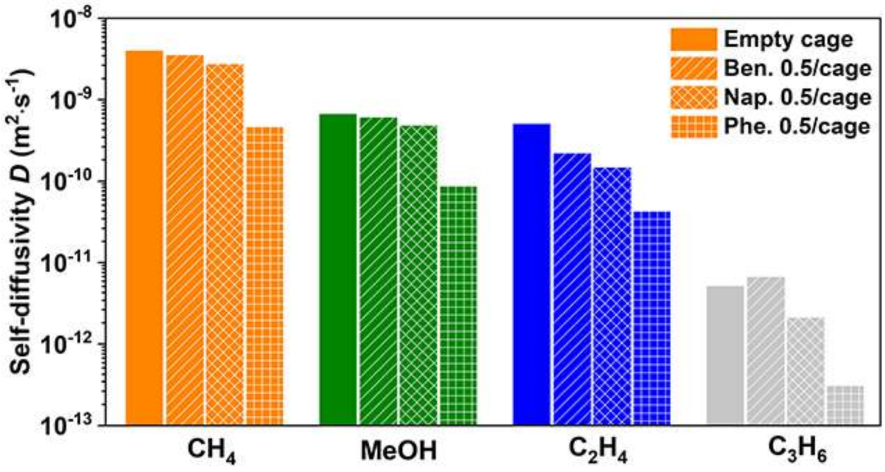

Supplementary Figure 16: The self-diffusivities of methane, methanol, ethylene and propylene at 723 K in AlPO4-34 zeolite with empty cage and loading with p-xylene, naphthalene and phenanthrene by MD simulations. The loading of methane, methanol, ethylene and propylene is 0.5 per cage. The loading of p-xylene, naphthalene and phenanthrene is 0.5 per cage.

Supplementary Note 7: Detailed results of reaction-diffusion simulations during MTO reaction

Concentration profile of retained carbonaceous species along the axial direction of catalyst bed during MTO reaction is shown in Supplementary Figure 17. The spatiotemporal distribution of reaction rate of methanol, HCP species and coke precursors during MTO reaction over SAPO-34-5, SAPO-34-12, SAPO-34-17 and SAPO-34-50 samples are shown in Supplementary Figure 18. The spatiotemporal distribution of diffusion flux of ethylene, propylene and C_{4+} are shown in Supplementary Figure 19.

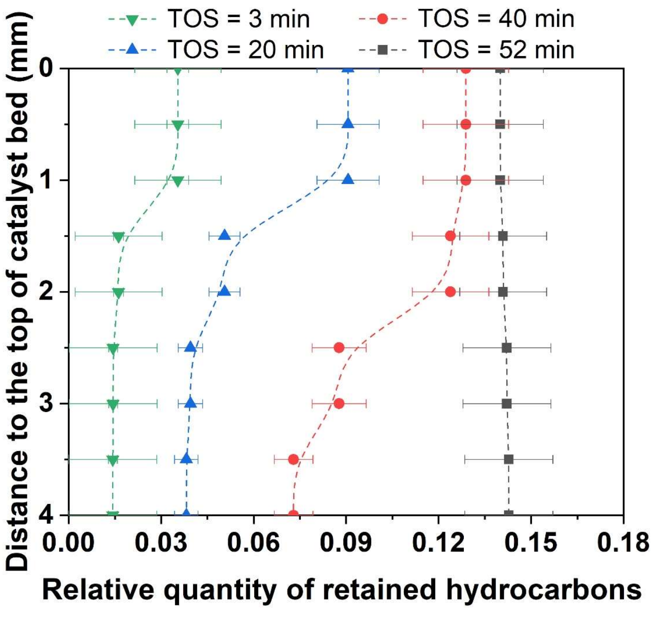

Supplementary Figure 17: Concentration profile of retained carbonaceous species along the axial direction of reactor during MTO reaction catalyzed by SAPO-34-12 zeolite sample. Simulation conditions: $T = 723$ K, $WHSV = 5.0 \pm 0.1$ $g_{MeOH} \cdot g_{zeo.}^{-1} \cdot h^{-1}$, partial pressure of methanol of 0.28 bar.

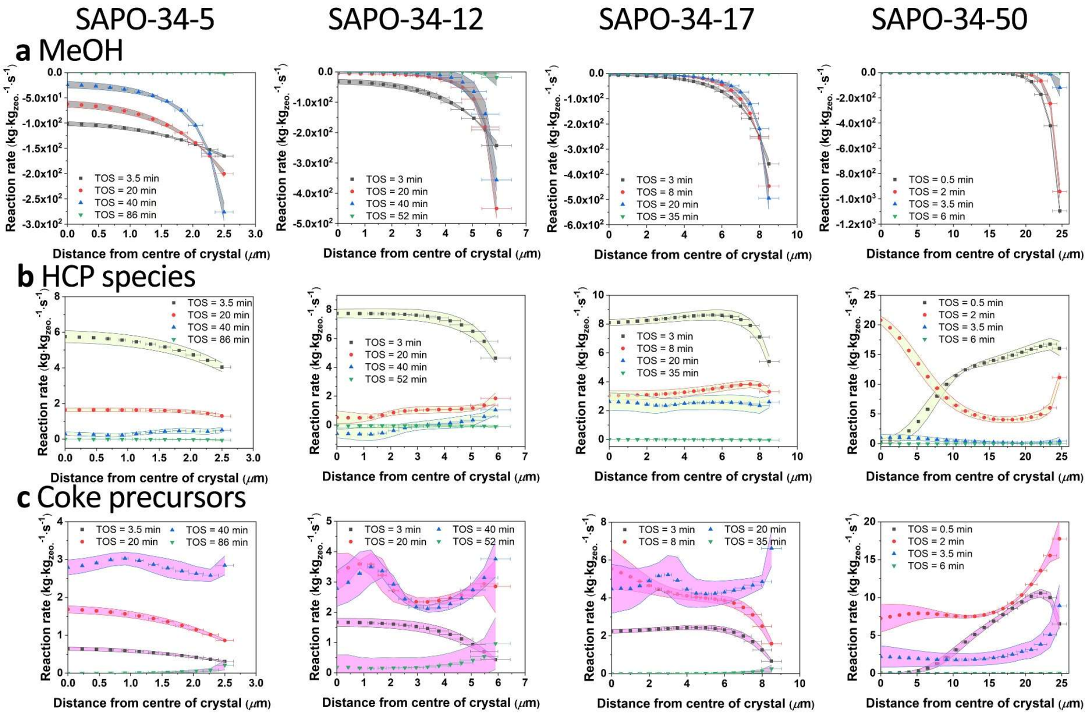

Supplementary Figure 18: The simulated spatiotemporal evolution of reaction rate of **a** methanol, **b** HCP species and **c** coke precursors in SAPO-34-5, SAPO-34-12, SAPO-34-17 and SAPO-34-50 crystals during MTO reactions at 723 K and *WHSV* of 5.0 ± 0.1 $g_{MeOH}\cdot g_{zeo.}^{-1}\cdot h^{-1}$. The quantity of acid sites is 1.00 ± 0.06 $mmol\cdot g_{zeo.}^{-1}$. The error band is standard error of simulation results. The sign of reaction rate of methanol is negative, which represents the consumption of methanol.

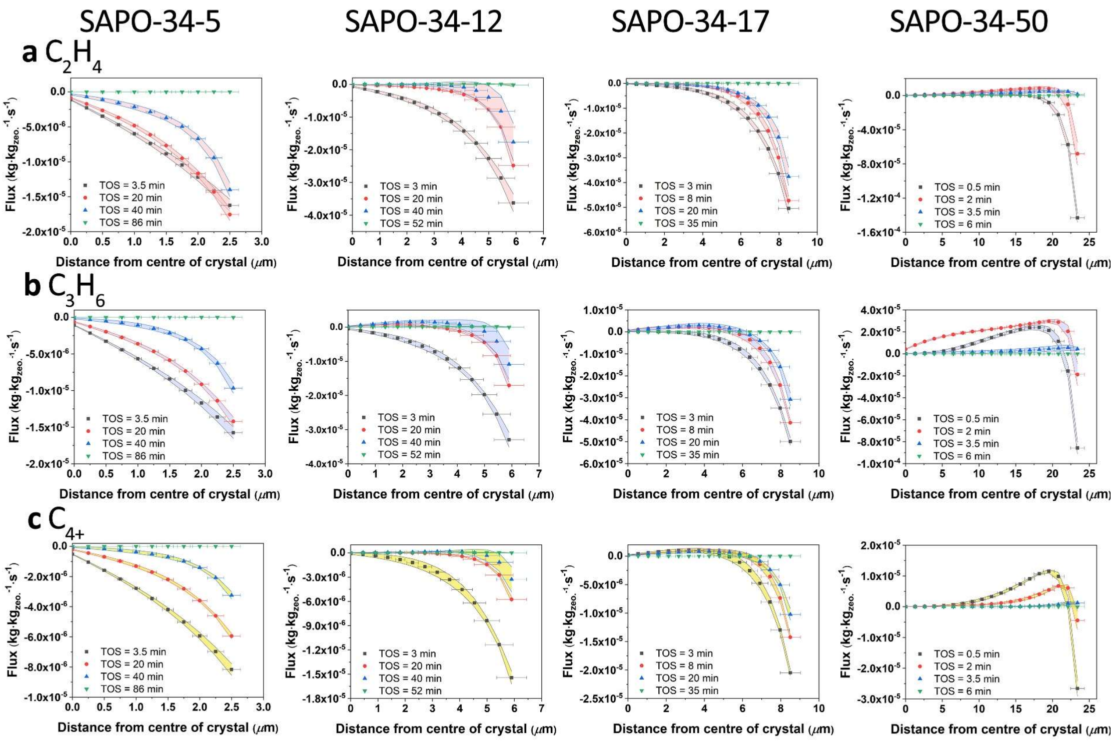

Supplementary Figure 19: The simulated spatiotemporal evolution of diffusion flux of **a** ethylene,

b propylene, **c** C_{4+} in SAPO-34-5, SAPO-34-12, SAPO-34-17 and SAPO-34-50 crystals during MTO reactions at 723 K and *WHSV* of 5.0 ± 0.1 $g_{MeOH}\cdot g_{zeo.}^{-1}\cdot h^{-1}$. The quantity of acid sites is 1.00 ± 0.06 $mmol\cdot g_{zeo.}^{-1}$. The error band is standard error of simulation results. The positive sign of diffusion flux represents chemicals diffusion toward crystal center, and negative sign of diffusion flux represents chemicals diffusion outward the crystal.

SUPPLEMENTARY REFERENCES

1. Yang, M. *et al.* A top-down approach to prepare silicoaluminophosphate molecular sieve nanocrystals with improved catalytic activity. *Chem. Commun.* **50**, 1845-1847 (2014).

2. Borodina, E. *et al.* Influence of the Reaction Temperature on the Nature of the Active and Deactivating Species During Methanol-to-Olefins Conversion over H-SAPO-34. *ACS Catal.* **7**, 5268-5281 (2017).

3. Hereijgers, B. P. C. *et al.* Product shape selectivity dominates the Methanol-to-Olefins (MTO) reaction over H-SAPO-34 catalysts. *J. Catal.* **264**, 77-87 (2009).

4. Gao, M. *et al.* A modeling study on reaction and diffusion in MTO process over SAPO-34 zeolites. *Chem. Eng. J.* **377**, 119668 (2019).

5. Li, H., Ye, M. & Liu, Z. A multi-region model for reaction–diffusion process within a porous catalyst pellet. *Chem. Eng. Sci.* **147**, 1-12 (2016).

6. Gao, M. *et al.* Direct quantification of surface barriers for mass transfer in nanoporous crystalline materials. *Commun. Chem.* **2**, 43-52 (2019).

7. Gao, S. *et al.* Cavity-controlled diffusion in 8-membered ring molecular sieve catalysts for shape selective strategy. *J. Catal.* **377**, 51-62 (2019).

8. Wang, C.-M., Wang, Y.-D. & Xie, Z.-K. Insights into the reaction mechanism of methanol-to-olefins conversion in HSAPO-34 from first principles: Are olefins themselves the dominating hydrocarbon pool species? *J. Catal.* **301**, 8-19 (2013).

9. Wang, S. *et al.* Polymethylbenzene or Alkene Cycle? Theoretical Study on Their Contribution to the Process of Methanol to Olefins over H-ZSM-5 Zeolite. *J. Phys. Chem. C* **119**, 28482-28498 (2015).

10. De Wispelaere, K., Hemelsoet, K., Waroquier, M. & Van Speybroeck, V. Complete low-barrier side-chain route for olefin formation during methanol conversion in H-SAPO-34. *J. Catal.* **305**, 76-80 (2013).

11. Wu, X. *et al.* Evolution of C–C Bond Formation in the Methanol-to-Olefins Process: From Direct Coupling to Autocatalysis. *ACS Catal.* **8**, 7356-7361 (2018).

12. Signorile, M., Bonino, F., Damin, A. & Bordiga, S. In Situ Resonant UV-Raman Spectroscopy of Polycyclic Aromatic Hydrocarbons. *J. Phys. Chem. C* **119**, 11694-11698 (2015).

13. Hemelsoet, K. *et al.* Identification of Intermediates in Zeolite-Catalyzed Reactions by In Situ UV/Vis Microspectroscopy and a Complementary Set of Molecular Simulations. *Chem. Eur. J.*

19, 16595-16606 (2013).

14. Van Speybroeck, V. *et al.* Mechanistic Studies on Chabazite-Type Methanol-to-Olefin Catalysts: Insights from Time-Resolved UV/Vis Microspectroscopy Combined with Theoretical Simulations. *ChemCatChem* **5**, 173-184 (2012).

15. M. J. Frisch *et al. Gaussian 09*, Revision B.01 edn (Gaussian, Inc., Wallingford CT, 2016).

16. Omori, N. *et al.* Understanding the Dynamics of Fluorescence Emission during Zeolite Detemplation Using Time Resolved Photoluminescence Spectroscopy. *J. Phys. Chem. C* **124**, 531-543 (2020).

17. Roeffaers, M. B. J. *et al.* Spatially resolved observation of crystal-face-dependent catalysis by single turnover counting. *Nature* **439**, 572–575 (2006).

18. Borodina, E. *et al.* Influence of the Reaction Temperature on the Nature of the Active and Deactivating Species during Methanol to Olefins Conversion over H-SSZ-13. *ACS Catal.* **5**, 992-1003 (2015).

19. Wong, Y. C., Ysselstein, D. & Krainc, D. Mitochondria–lysosome contacts regulate mitochondrial fission via RAB7 GTP hydrolysis. *Nature* **554**, 382–386 (2018).

20. Qi, Q. *et al.* A H-bond strategy to develop acid-resistant photoswitchable rhodamine spirolactams for super-resolution single-molecule localization microscopy. *Chem. Sci.* **10**, 4914-4922 (2019).

21. Ghysels, A. *et al.* Shape-Selective Diffusion of Olefins in 8-Ring Solid Acid Microporous Zeolites. *J. Phys. Chem. C* **119**, 23721-23734 (2015).

22. Dai, W., Scheibe, M., Li, L., Guan, N. & Hunger, M. Effect of the Methanol-to-Olefin Conversion on the PFG NMR Self-Diffusivities of Ethane and Ethene in Large-Crystalline SAPO-34. *J. Phys. Chem. C* **116**, 2469-2476 (2012).
